# Fragmented Governance, Shared Landscapes: Policy and Functional (In)Coherence Insights from the Great Limpopo Transfrontier Conservation Area

**DOI:** 10.1007/s00267-025-02309-9

**Published:** 2025-11-17

**Authors:** Ephraim Mpofu, Marianne Penker, Walter Musakwa, Verena Radinger-Peer, Katharina Gugerell

**Affiliations:** 1 Department of Landscape, Water and Infrastructure, Institute of Landscape Planning, BOKU University, Vienna, Austria; 2 Doctoral School Transitions to Sustainability, BOKU University, Vienna, Austria; 3 Department of Economics and Social Sciences, Institute for Sustainable Economic Development, BOKU University, Vienna, Austria; 4 Department of Geography, Environmental Management and Energy Studies, University of Johannesburg, Johannesburg, South Africa; 5 Department of Landscape, Water and Infrastructure, Institute of Landscape Development, Recreation and Conservation Planning, BOKU University, Vienna, Austria

**Keywords:** Transfrontier conservation areas, Great Limpopo, Conservation, Policy, Landscape governance

## Abstract

Transfrontier Conservation Areas (TFCAs) have been operating under unsolved theoretical puzzles related to policy coherence and practical coordination. In particular, the mismatch between national policies and ground operations warrants a thorough policy and functional coherence assessment, which remains underexplored in TFCAs. Through a qualitative comparative analysis of strategic park policy documents and twenty key informant interviews, this study examines the extent of policy and functional coherence between Kruger and Gonarezhou National Parks within the Greater Limpopo TFCA (GLTFCA). The study applied a theme-based coding method to assess alignment across functional policy domains. Our findings reveal a moderate policy alignment (3.6 out of 5) with strong coherence in themes aligned to international frameworks and global norms. In contrast, themes related to governance, institutional efficiency, and transboundary cooperation showed the weakest coherence, highlighting implementation gaps and fragmented accountability. These weaknesses correspond closely to key coordination dimensions, particularly those related to institutional alignment and knowledge-sharing mechanisms. These findings underscore that while policy intent aligns, functional coherence is constrained by disparities in power, institutional capacity, the complexities of legal pluralism, and fragmented coordination mechanisms. The findings underscore that aligning policy intent is insufficient without addressing functional coherence and call for greater attention to political, institutional, and legal asymmetries in TFCAs. This research also contributes to ongoing efforts for policy harmonization within the GLTFCA by offering a practical, theme-based method for diagnosing alignment gaps and overlaps across strategic conservation policies. It provides empirical insights into the current disconnect between policy provisions and operational realities, highlighting critical areas in need of immediate attention and resource allocation.

## Introduction

The transboundary nature of natural resource challenges, such as biodiversity loss and habitat degradation, has underscored the need for multi-actor collaboration (Petursson et al.; Vedeld and Vatn 2013; Siangulube et al. 2023). Such challenges necessitate coordinated policy responses among neighboring countries to address the complex challenges of sustainable resource management on a larger scale. Concepts such as ‘bioregionalism’, which asserts that boundaries of protected areas should be drawn around ecosystems, rather than following political boundaries (Wolmer 2003; Wearne et al. 2023) have contributed to the emergence of new conservation approaches such as the transfrontier conservation areas (TFCAs). However, since conception, TFCAs have been operating under unsolved theoretical puzzles related to policy coherence and practical coordination (Linell et al. 2019).

While TFCAs have received considerable endorsement from the international community, the effective implementation and sustenance of these agreements (treaties) at all governance levels comes with considerable friction and potential conflict (Spierenburg and Wels 2006; 2008). In particular, the mismatch between national policies and ground operations has led to shortcomings in policy execution, resulting in “free-riding” of common pool resources (Ostrom 2005; Linell et al. 2019). Achieving alignment both within and between countries has remained a challenge in many regions, owing to inconsistent legal and institutional frameworks and differing resource management strategies (Petursson et al. 2013; Schoon 2013). Previous research has identified such instances as requiring thorough policy and functional coherence assessment (Howlett et al. 2017; Retief et al. 2023). While both policy and functional coherence are essential to ensure effective institutional performance, their synergy facilitates a comprehensive overhaul of assessment, bridging the gap between theoretical frameworks and practical implementation. Policy coherence focuses on alignment and consistency of policy goals, objectives, and activities across different sectors and levels (Koff et al. 2016; Dombrowsky et al. 2022). Functional coherence ensures the coordination of institutional mechanisms, administrative structures, and processes (Dombrowsky et al. 2022). While such assessments remain underdeveloped in TFCAs, despite their establishment through national-level formal agreements, the realities of implementation often diverge at local level (Muboko 2017; Retief et al. 2023).

These inconsistencies in policies have always been inherent among national policies, hence the need for thorough policy coherence assessment before harmonization (Büscher and Schoon 2009; Schoon 2013). This further facilitates effective policy diffusion and harmonization (Marsh and Sharman 2009; Howlett et al. 2017) providing insights into how policies from one country can be adapted and implemented in another, identifying both enabling factors and barriers to cross-border collaboration. While high-level policies are typically considered for harmonization, lower-level policies that guide landscape-level or ground implementation often face challenges, as strategic alignment happens mostly at the national level (Spierenburg et al. 2008; Büscher and Dressler 2012). This is evident in TFCAs as the treaties are signed at a national level, but local actors (communities, national parks) often find it difficult to collaborate effectively (Linell et al. 2019). Bottom-up policy coherence is also essential in addressing local actors’ needs, as ground realities reveal practical challenges that are often overlooked by centralized governance structures (Sayer et al. 2013; Imperial 2021). This approach increases actor buy-in by ensuring that field practitioners recognize the value of their operational expertise and local knowledge in shaping policy. Therefore, the synchronization of ground operations is vital, not solely at high-level, but also because they serve as practical mechanisms through which policies are enacted.

This research focuses on two of the GLTFCA countries (South Africa and Zimbabwe). It argues that while the need for policy harmonization within the GLTFCA is recognized (Child et al. 2012; Muboko 2017; Linell et al. 2019; Mpofu et al. 2023), assessing policy and functional coherence remains underexplored. It emphasizes that effective transboundary conservation depends on aligning practical operations alongside high-level policy objectives to translate intentions into actionable outcomes. While each country does not need to adopt a uniform management style, establishing areas of coordination and collaboration through policy coherence is essential for the efficient management of shared natural resources. Little empirical work has been done to compare national park-level conservation policies between the two countries within a similar TFCA. Existing literature focuses on high-level policy research and ecological outcomes of TFCAs (Linell et al. 2019; Chitakira et al. 2022; Retief et al. 2023). Literature suggests that both policy and functional incoherence are a product of aligned institutional frameworks and knowledge systems, which allow actors to operate based on shared rules, goals, and information flows (Pahl-Wostl 2009; Cejudo and Michel 2017). Shawoo et al. (2023) further indicated that effective coordination also requires active collaboration among diverse actors, with integrative stakeholders bridging institutional and sectoral divides to harmonize strategies and actions (Dombrowsky et al. 2022). Coordination is therefore nested within the concept of coherence (Voyer et al. 2020). Building on this, Dombrowsky et al. (2022) operationalize coordination through five dimensions: institutions, actors, integrators, knowledge, and action situations, which provide a functional lens to assess how coordination supports or undermines policy coherence. While vertical coordination challenges are apparent, our study primarily focuses on horizontal coordination driven by the growing debates on collaboration challenges among national parks within the GLTFCA. It seeks to address three questions: (i) To what extent are strategic policy goals and objectives aligned between Kruger- and Gonarezhou National Parks (ii) Which key areas exhibit the highest and lowest levels of coherence in Policy between the two national parks (iii) Which dimensions of coordination (according to Dombrowsky et al. 2022) have the most impact on functional coherence? While the study seeks to identify the drivers of policy and functional (in)coherence (Research Questions 1 and 2), our study further investigates the primary dimensions through which this incoherence manifests (Research Question 3), following the work of Dombrowsky et al. (2022). Therefore, coordination in this context is viewed through five empirically grounded dimensions: institutions, actors, integrators, knowledge, and action situations.

This approach enables a systematic assessment of the specific mechanisms and dimensions through which coordination breaks down, moving beyond the generic descriptive focus on ‘what’ issues to uncover the underlying processes driving incoherence. Our research contributes to ongoing debates on policy harmonization in TFCAs, not only through empirical insights on the discrepancies between national-park level policies, which are often overlooked, but also by advancing the conceptual understanding of coordination within policy coherence debates - an area that remains underexplored in TFCA scholarship.

### Literature Review: Policy and Functional (In)Coherence in TFCAs

The establishment of TFCA policies is undeniably influenced by the national legislative policies (Wolmer 2003; Hanks and Myburgh 2015). National conservation authorities are primarily responsible for the formulation and enactment of policies that pertain to conservation at the national level. However, these authorities work within the broader regional and global frameworks such as the Southern African Development Committee (SADC) TFCA program, Convention on Biological Diversity (CBD), United Nations Sustainable Development Goals (SDGs). This, however, poses a challenge to the micro-level policies as higher-level strategic objectives often lack direct relevance to micro-level policies (Roux et al. 2016; Carolus et al. 2018). National-level policies in most cases, are too broad to address the specific, localized needs of national parks and communities within TFCAs (Duffy 2006; Retief et al. 2023). With regards to Southern Africa, many TFCAs operate under a highly centralized governance model, where key decisions are made at the national or intergovernmental level, with limited meaningful input from local actors (Spierenburg et al. 2008; Thakholi et al. 2024). As a result, local actors bear the burden of navigating a complex landscape with conflicting policies, compromising their ability to implement effective conservation measures.

Issues of sovereignty have often added to these challenges, creating the challenge of policy fragmentation, where each country is bound to its laws with little to no consideration of the other (Schoon 2013; Muboko 2017; Retief et al. 2023). While policy in(coherence) within TFCAs manifests as a phenomenon of policy fragmentation, contradicting national park regulations, asymmetrical stakeholder engagement, and poor coordination across and within national borders, it reflects deeper political and legal complexities, primarily rooted in power asymmetries and the persistence of legal pluralism (Benda-Beckmann 2001; Ros-Tonen et al. 2018; Siangulube et al. 2023). Drawing on political economy perspectives, in Southern Africa, policy in(coherence) is also attributed to colonial legacy and legal pluralism (May et al. 2006; Rautenbach 2010; Ubink et al. 2021). Legal pluralism entails the coexistence of multiple legal systems (formal state law, customary law, international conventions, and informal governance norms) within the same social field. This provides a structural condition for power asymmetries, which then cascades into policy in(coherence).

The implementation of fragmented policies in TFCAs such as Kavango–Zambezi (KAZA), Great Limpopo, and Kgalagadi TFCAs has posed significant challenges, emphasizing the urgent need for policy harmonization (Chitakira et al. 2022; Retief et al. 2023). Fragmented policies lack coordination in various aspects of institutional operations. This has been evident in aspects of resource usage where some parties exploit resources without contributing equitably to their conservation. For example, the KAZA TFCA experiences inconsistencies in fishing regulations across national borders (Linell et al. 2019). While countries like Botswana enforce seasonal restrictions to support fish population recovery, neighboring states permit year-round fishing, undermining collective efforts to sustainably manage shared aquatic resources. These policy discrepancies enable some countries or resource users to benefit disproportionately, exploiting resources at the expense of others who adhere to stricter conservation measures. Another notable example is with hunting policies. In certain countries within the TFCAs, hunting is permitted as a regulated activity for economic and conservation purposes in some countries, while other member states enforce strict no-hunting policies (Tchakatumba et al. 2019; Botha and Murphree 2022). This has been reported in Kgalagadi and KAZA where Botswana has historically adopted a ban on hunting, particularly elephants, to protect its wildlife populations, while Namibia and Zimbabwe allow regulated trophy hunting as a means of generating revenue for conservation and local communities (Linell et al. 2019; Tchakatumba et al. 2019; Mbaiwa and Hambira 2023). Discrepancies in aspects of law-enforcement in national park policies have also resulted in the overflow of animals into countries with stricter protections. This has been observed in Kruger National Park (KNP) and Gonarezhou National Parks (GNP), where wildlife, particularly elephants, has migrated from Mozambique’s Limpopo National Park, which struggles with high poaching rates (Ntuli et al. 2021; Henley et al. 2023). The disproportionate influx of animals into KNP and GNP often leads to overpopulation, resulting in overgrazing and habitat degradation, and a subsequent increase in HWC (Louw et al. 2021; Hawker 2023). Such misalignments create tensions and competitive dynamics between actors, weakening cross-border resource management (Botha and Murphree 2022).

While some cases can be tied to policy fragmentation, some functional coordination mainly stem from institutional inefficiencies. For instance, while collaborative law enforcement may necessitate the involvement of various departments, such as immigration and border control, critical issues like the establishment of standardized communication protocols among national parks within TFCAs remain unresolved (Chiutsi and Saarinen 2017, 2019; Botha and Murphree 2022). Additionally, standardized approaches to wildlife censuses, particularly for species like elephants that traverse multiple national parks within TFCAs, remain underdeveloped. Uncoordinated census across neighboring countries is ineffective if one nation conducts a census while others do not, given that animals freely roam across borders. These issues in turn undermine the goals and existence of a TFCA in the first place (Schenning 2019). While specific issues vary across each TFCA, these challenges underscore key gaps and reveal opportunities for collaborative management and operational strategies that could enhance effectiveness both within individual national parks and across the TFCA as a whole.

## Materials and Methods

This research employed a mixed-methods approach combining qualitative and quantitative analysis in comparing national park policies and expert interviews. The study is grounded in Comparative Policy Analysis (CPA) (Benson and Jordan 2011) which enables structured comparisons across policies by examining core dimensions such as objectives, implementation mechanisms, outcomes, and relevant contextual factors. Additionally, our framework integrates insights from Policy Transfer and Diffusion Studies (Dolowitz and Marsh 2000) to explore how policies from distinct contexts are adapted or transferred to others. These theoretical perspectives facilitate a systematic comparison of the policies from GCT and KNP to identify gaps and collaborative opportunities within the GLTFCA. Combining this approach with semi-structured interviews allowed for triangulation, ensuring that findings are robust and well-rounded. Two dimensions of coherence were examined which include policy coherence and functional coherence follows the framework established by Dombrowsky et al. (2022) where functional coherence is achieved when: (i) All governance functions are formally assigned, ensuring no gaps exist where responsibilities remain undefined (ii) Governance functions are clearly divided and consistently applied, so each actor understands their role and mandate, creating a strong foundation for coordination (iii) Coordination mechanisms are in place for shared governance functions, ensuring that all relevant actors are aware of each other and understand their roles in collaboration, thereby facilitating effective coordination and problem-solving. Functional coherence was further branched based on process or governance level and assessed based on certain coordination or coherence aspects.

### Document Analysis

Policy selection included relevant policies addressing conservation, objectives, and implementation actions in both KNP and GNP (National-Park Strategic/Management Plans). Up-to-date versions of the policies were selected to enhance the relevance and applicability of the findings. Document analysis was executed to identify objectives of various conservation activities within the policies of the two countries. To ensure that the comparison was robust and comprehensive, deductive coding based on a combination of existing thematic areas from the policy documents and relevant literature was used to develop universal themes. These themes represent broad policy categories to which both policies can be benchmarked and compared from rather than focusing on policies categorized by their specific themes. The objective was to identify overarching areas that are typically addressed in the management of protected areas, ensuring all relevant aspects of park operations were captured in the analysis. Specific themes within the two policies reflected commonality but presented different terminology, scope, and detail. Their framing aligns with those frequently observed in the literature, such as the classifications outlined by (Voyer et al. 2020). Once the universal themes were defined (Table 1), the individual objectives from each park’s policy document were grouped under these respective themes. MAXQDA, a qualitative data analysis software, facilitated the coding and classification of objectives based on thematic content analysis. Hierarchical coding of policy objectives and subsequent activities was used, where broad categories were divided into more specific, finer categories to identify key patterns and relationships. This allowed for the categorization of objectives systematically and ensured that all objectives fit into their appropriate themes.

**Table 1:** Deductive codes and subcodes to guide in objective categorization based on foundational literature in conservation and its mandates

Code	Subcode	Literature
1. Conservation and environmental management:	-managing ecosystems -biodiversity preservation and protection -addressing environmental threats -natural resource management	(Mace 2014; Voyer et al. 2020)
2. Community engagement and socio-economic development:	-community involvement and communication -capacity-building -promoting economic benefits from conservation activities.	(Chitakira et al. 2012; Chiutsi and Saarinen 2017)
3. Land use, regional planning, and resource management:	- land use -regional planning -institutional arrangements -peripheral land management.	(Hanks and Myburgh 2015; Mpofu et al. 2025)
4. Connectivity and transboundary cooperation:	-cross-border cooperation and communication -disease management at the interface of wildlife and human activities -transboundary corridors.	(Wolmer 2003; Hanks and Myburgh 2015)
5. Commercial strategy and resource utilization:	-commercial models -branding -stewardship -resource management to support conservation financially.	(Büscher 2013; Linell, Sjöstedt and Sundström 2019)
6. Corporate governance and institutional efficiency:	-internal functioning, and governance structures of conservation programs. -compliance and enforcement	(Voyer et al. 2020; Retief et al. 2023)
7. Infrastructure and resource management:	-physical infrastructure needed for conservation, tourism, and resource management efficiency.	(Spierenburg and Wels 2006; Büscher 2013)
8. Cultural heritage and historical conservation:	-recognition and preservation of cultural heritage	(Gandiwa et al. 2014; Mpofu et al. 2025)
9. Monitoring and evaluation:	- tracking implementation, -adjusting strategies based on data -maintaining decision support systems.	(Linell et al. 2019; Voyer et al. 2020)
10. Communication and awareness	-information flow and distribution -knowledge co-creation	(Mace 2014; Voyer et al. 2020)

After coding the policy objectives and putting them in respective themes, comparison of the objectives was executed. An analytical framework (Table 2) based on the previous work of Gugerell et al. (2020) and Dolowitz and Marsh (2000) was employed. To enhance the validity of our qualitative content analysis, we additionally employed a scoring system method, which provides a systematic and reproducible framework for comparing subjective (qualitative) data (Fakis et al. 2014; Halevi Hochwald et al. 2023). This method has been used in similar research to bridge qualitative and quantitative methods (England 2021; Siangulube et al. 2023). The approach also allowed for the establishment of an alignment scale (Table 3) for ease of interpretation. Quantifying qualitative alignment can help to illuminate specific areas for improvement. However, while we acknowledge that the scoring system can be subjective (Fakis et al. 2014) and that the method of quantifying qualitative data is at its early development stages (Halevi Hochwald et al. 2023), we integrated it as a supplementary tool alongside the well-established qualitative content analysis, which also contributes to methodological advancements in policy comparison.

**Table 2:** Alignment criteria for comparison of the objectives: analytical framework

Alignment criteria	Description
Similarity of goals	Are the goals stated similarly or are they diverging?
Consistency in objectives	Do the policies of Zimbabwe (GNP) and South Africa (KNP) have common or shared objectives (e.g., anti-poaching, species protection)?
Coherence in implementation strategy	Are the strategies to achieve the goals consistent (e.g., patrols, community involvement)?

**Table 3:** Policy alignment scale from 1 (low) to 5 (high)

#	Alignment	Description
0	No alignment	One Park lacks an objective or strategy in the thematic area.
1	Minimal alignment	Very little overlap in context of the objectives or implementation actions.
2	Low alignment	Policies have some objectives in common but are not sufficiently aligned in terms of actions or scope.
3	Moderate alignment	There is a clear overlap in objectives, but the scope or scale of actions varies slightly.
4	High alignment	The parks have identical or near-identical objectives and implementation strategies.
5	Complete alignment	The parks have fully aligned objectives and strategies, with no discernible differences in action, scope, or implementation methods.

Scores for each theme were determined by aggregating objective-level scores into thematic indices. These thematic indices were subsequently combined to generate an overall index (alignment) score between the two policies (See *formula 1* and *2*). This approach aligns with methodologies used by Wendling et al. (2022) and Sachs et al. (2022), which employ similar principles to systematically assess and integrate multiple indicators across themes. It allowed for a standardized comparison of alignment across all themes and ensured that the final scores reflected both the presence and extent of alignment between the parks.

$$({formula}1)\,O\mathrm{verall\; Theme\; Alignment\; Score}=\frac{\sum \mathrm{Alignment\; Scores\; per\; Objective}}{\mathrm{Total\; Number\; of\; Objectives}}$$

$$({formula}2)\,\mathrm{Overall\; Extent\; of\; Policy\; Alignment}=\frac{\sum \mathrm{Alignment\; Scores\; per\; Theme}}{\mathrm{Total\; Number\; of\; Themes}}$$

### Semi-structured Interview Analysis

To determine functional coherence, semi-structured interviews with twenty key informants were conducted (Table 4). Snowball sampling was employed to identify suitable interviewees, leveraging participants’ networks to reach key informants with relevant expertise. Interviews allowed for the collection of in-depth, ground-level insights that are not accessible through policy document analysis alone. The analysis of interview data aimed at identifying and understanding operational challenges faced on the ground by both national parks within GLTFCA. These practical challenges reveal critical areas where policy evolution can foster improved alignment and operational efficiency across borders. The participants were selected based on their direct involvement in the national parks within the GLTFCA, with representation from both the national and local (national-park level) governance levels.

**Table 4:** Institutional affiliation and jurisdictional distribution of key informants (20) interviewed for the study

Organization/actor	Position(s)	Number (gender)	Stakeholder category	Jurisdictional level	Country
Gonarezhou Conservation Trust (GCT)	Director, Area Managers, Community Liaison Officers	4 (Males)	National Park	District	Zimbabwe
Kruger National Park (KNP)	GEF-7 Regional Coordinator, Park Ranger	2 (Males)	National Park	District	South Africa
Department of Agriculture, Land Reform and Rural Development (DALRRD)	Senior Manager, Development Planning Support	1 (Female)	Ministry	National	South Africa
Department of Forestry, Fisheries and the Environment (DFFE)	Director, Transfrontier Conservation Areas	1 (Female)	Ministry	National	South Africa
Ministry of Environment, Water and Climate (MEWC)	Policy and Governance Expert	1 (Male)	Ministry	National	Zimbabwe
Makuleke Community Game Reserve	Community Representative	1 (Male)	Community Conservation	Local	South Africa
Bende-Masisi Community Structure	Community Leader	1 (Male)	Community Structure	Local	South Africa
Malipati Development Trust	Community Representative	1 (Male)	Community Conservation	Local	Zimbabwe
Malilangwe Wildlife Reserve	General Manager & Neighbor Outreach Programme Coordinator	1 (Male)	Private Conservation	Local	Zimbabwe
Greater Kruger Private Sector	Kruger to Canyons Biosphere Manager, Timbavati Game Reserve CEO	2 (Males)	Private Conservation	Local	South Africa
GLTFCA International Coordinator	International Coordinator	1 (Male)	Transboundary Governance	International	Zimbabwe
GLTFCA National Coordinator (Zimbabwe)	National Coordinator	1 (Male)	Transboundary Governance	National	Zimbabwe
Zimbabwe Parks and Wildlife Authority (ZimParks)	Director, Transfrontier Conservation Affairs	1 (Female)	Parks Authority	National	Zimbabwe
South African National Parks (SANParks)	Senior Official	1 (Female)	Parks Authority	National	South Africa
Chiredzi Rural District Council (RDC)	Chief Executive Officer	1 (Male)	Local Government Authority	District	Zimbabwe

The respondents represent a diverse cross-section of actors engaged in the governance and implementation of conservation policies in the GLTFCA

Ethical clearance was granted by the University of Johannesburg (2022-10-03/Musakwa), and all applicable regulations governing the operations within the national parks were observed. Research permits were subsequently provided for both KNP (SANParks research-SS798) and GNP (Research permit 151/43/P/REND). The interviews were recorded, and each interviewee was assigned a unique identifier code to ensure data protection. These interviews were conducted in South Africa and Zimbabwe with relevant actors having a key bearing in KNP and GNP, respectively. MAXQDA was also used for hierarchical coding of the interview statements, where operational challenges formed the basis of the coding.

The themes identified in the initial stage of policy analysis were retained as they encompass the full spectrum of conservation dimensions within the GLTFCA. The interview statements were first assigned to specific challenges based on the coding scheme. These specific codes (subcodes) were further categorized to ensure they fit within the broader, overarching themes that represent the primary focus of our analysis. This involved merging sub-codes with high overlap in addition to those that exhibit the same concept. The documenting of reasoning behind this coding decision was also done using memos to ensure clarity on how the coding decisions were made. Further reassessment was done to ensure that each statement was consistently and correctly coded. The coded data was then exported in Excel format (entire transcript) and cleaned for further manipulation in Tableau, a powerful data visualization tool that enables the transformation of complex data into meaningful visual insights. This was because, at this stage, our analytical framework demanded numerous angles of results visualization (e.g., themes, coordination aspects, distribution in aspects per country, governance level). This enhanced the depth, clarity, and interpretability of the results. While traditional methods of results presentation fail to capture complex relationships, patterns, and interconnections within the raw data, we leveraged our expertise in data science concepts such as data structuring, manipulation, and visualization to present our results in Tableau.

## Results

### Policy Coherence

**Fig. 1:**
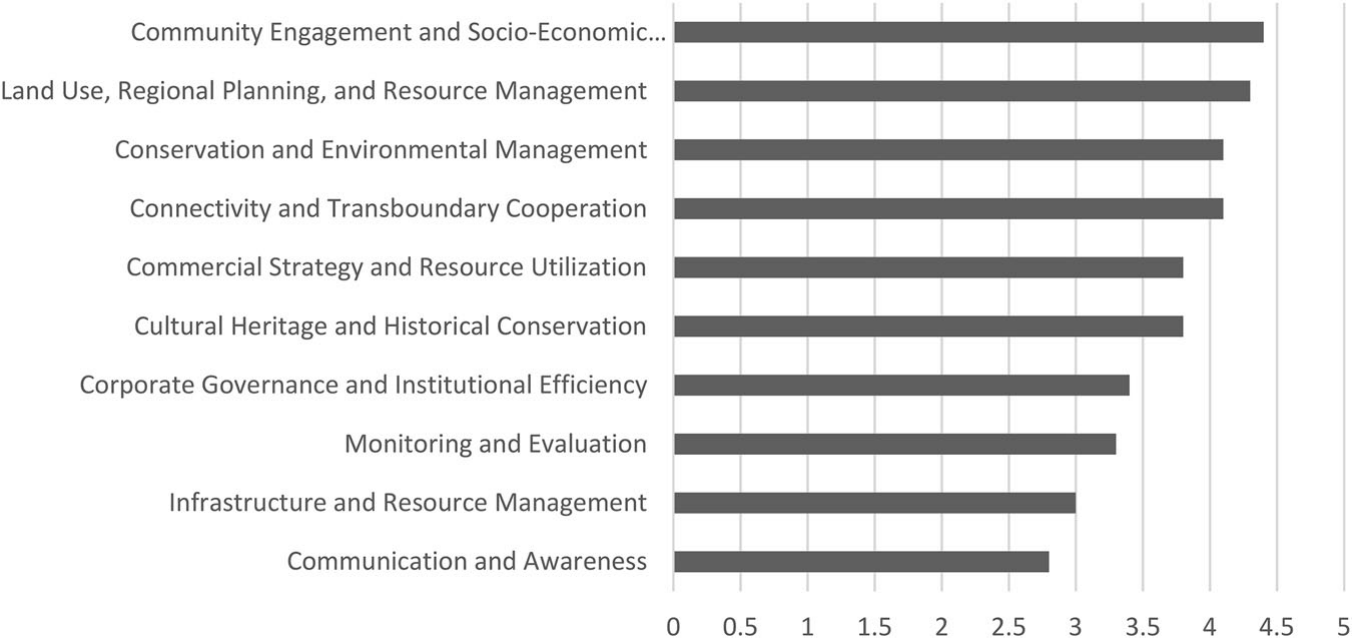
Alignment scores for each theme within National Park policies. Each theme score represents the cumulative alignment of individual policy objectives, while the overall policy alignment is derived from the aggregation of all theme scores

The results of the policy alignment analysis reveal varying degrees of coherence across different thematic areas between the two national parks in the GLTFCA. Ten themes were extracted from the policies, with variations being moderate rather than extreme Fig. 1.

$${\rm{Overall\; Extent\; of\; Policy\; Alignment}}=\frac{\sum 2.8;3;3.3;3.4;3.8;3.8;4.1;4.1;4.3;4.4}{10}={\bf{3}}{\boldsymbol{.}}{\bf{6}}$$

“Community Engagement and Socio-Economic Development”, “Land Use, Regional Planning, and Resource Management”, and “Conservation and Environmental Management” exhibit the highest levels of alignment, each scoring over the threshold of 4 on the alignment scale. Both parks demonstrate strong alignment in the socio-economic development overarching goals and objectives, despite varying methods, with KNP focusing on broader regional initiatives while GNP takes a more localized approach (Supplementary Appendix 4). The second-highest alignment reflects strong policy alignment, with both parks pursuing convergent objectives through complementary yet distinct approaches. KNP focuses on structured policy frameworks and formal planning processes, while GNP emphasizes pragmatic action-driven, on-the-ground implementation. The two parks also share several key conservation priorities with a minor gap, where KNP’s focus on other things like pollution prevention, which is indirectly addressed in GNP’s objectives.

The second category of themes with middle-tier score points (alignment score >=3 and 4<=) includes “Commercial Strategy and Resource Utilization”, “Cultural Heritage and Historical Conservation”, “Corporate Governance and Institutional Efficiency”, and “Monitoring and Evaluation”. Our results show a fairly strong alignment with regard to sustainability, heritage conservation, governance, and monitoring. GNP emphasizes cross-border tourism development, while KNP leans towards park sustainability, strategic marketing, and partnerships. Partnerships with the community are also important when it comes to governance in the GNP, as it focuses on direct staff development and strengthening relationships at the community level, while KNP focuses on SANParks alignment and broader institutional frameworks. A more community-centered approach is highlighted in the context of GNP, as their approach in cultural heritage conservation focuses on awareness-building, such as documentation, feasibility studies, and consultation regarding cultural sites. In comparison, KNP takes a more formalized strategy for cultural site management, focusing on archeological excavation and staff awareness regarding cultural heritage. Notably, when it comes to monitoring GNP appears to focus more on short-term, responsive actions, such as human-wildlife conflict (HWC) management, while KNP integrates long-term monitoring and broader system improvements, such as program evaluation, research contributions, and adaptive management.

The lowest scores are observed in “Infrastructure and Resource Management” and “Communication and Awareness”. The key difference lies in who they target, how they engage, and through which channels. KNP is policy-driven, while GNP is community-driven. This level of alignment suggests that both parks have several gaps in the alignment of their priorities. The policy alignment score between the two policies, cumulative of individual themes indicates a moderate alignment (3.6) across the policies of the two national parks. While there is some alignment in the policy intent and objectives, significant discrepancies remain evident in operational and thematic focus.

### Functional Coherence (Policy Implementation)

In functional coherence, “Corporate Governance and Institutional Efficiency” emerges as the most prominent, exhibiting the most coherence-related challenges. Based on the interview data, the challenges are primarily centered around the institutional structures, inefficiency in the bureaucratic system, and prolonged policy Implementation. Unproductive meetings emerged as a key concern, frequently highlighted in relation to “Corporate Governance and Institutional Efficiency”. One of the respondents cited, “*People talk about these meetings, spend a lot of money and workshops, but at the end if you check what we achieved… you don’t see results*.” Such sentiments were prevalent at all governance levels, with further results pointing towards complex institutional structures. This was reflected in statements like “*Now you have this really complicated structure where you’ve got the JMB and then you got the national level committees, then you multiply those out in every TFCA*”. Related comments were often reiterated, solidifying the impact of institutional structures, which are deemed to be in disarray. The prolonged duration of implementing the originally proposed policies was frequently highlighted, seemingly contributing to a sense of fatigue and obsolescence among institutional stakeholders. While a minority of participants cite resource constraints as the cause of implementation delays (e.g., Secretariat -human resource), most respondents attribute the issue to obsolete evaluation mechanisms, impractical policies, and the absence of fundamental frameworks pertinent to landscape-level cooperation rather than higher-level policy agreements. Significant issues also emerge in “Connectivity and Transboundary Cooperation”. Respondents highlight varying commitments, absence of practical formalities on the ground, and absence of a crossing border facility and a functional corridor. The absence of a functional corridor was highly emphasized, especially from the Zimbabwean side. Respondents alluded to this issue as a major concern within the GLTFCA, highlighting the dysfunction of the Sengwe-Tshipise corridor which connects GNP to other GLTFCA national parks as a limitation to the TFCA. One respondent from Zimbabwe mentioned, “*We still don’t have a border crossing.…first ingredient for this TFCA to actually work is that we need a functional link between KNP and GNP because, without the Sengwe-Tshipise corridor, you’ve actually got no participation of Zimbabwe in the TFCA”*. Operations such as fire management and cross-border security were also among the grappling issues constraining coherence between KNP and GNP. Officials at GLTFCA landscape level indicated that informal practicalities end up being employed, often resorting to personal relationships to manage fire and poaching that go beyond borders. The absence of well-defined policies and strategies to combat critical threats like fire and poaching is a major deficiency, particularly given their direct impact on the core mission of the national parks.

“Community Engagement and Socio-Economic Development” also emerged as one of the main challenges. The lack of effective channels for communication and feedback is a major concern with several actors in the GLTFCA landscape echoing each other. Several comments reflect perspectives shared by participants across all levels of governance (i) “*The communications plan is still not in place… we do not have a mandated way of communicating properly*” (ii) “*there’s no place for a community member to go and find the livelihood plan, tourism development plan, and the cross-border security plan*”. All these issues point to a major constraint in the communication framework and how information is not being effectively disseminated to the communities. Most community members are not aware that they are part of the GLTFCA, hence some respondents indicated “*awareness and advocacy is also one of the key important aspects in terms of transmitting information to the ground*”, advocating for meaningful enlightenment and empowerment through awareness. However, other themes such as “Infrastructure and Resource Management”, “Land Use, Regional Planning, and Resource Management”, “Conservation and Environmental Management”, “Commercial Strategy and Resource Utilization”, remain mediocre, except for “Cultural Heritage and Historical Conservation,” which showed fewer challenges in comparison. Furthermore, most themes (six out of ten) exhibited challenges primarily at the process level rather than the outcome level.

Results also show that the distribution of these themes in relation to the aspect of coordination varies. From the five presented coordination aspects, “coordination via knowledge” is broadly distributed, having impact across all themes. While this is the case, the theme “communication and awareness” carries the most impact when it comes to this coordination aspect, followed by “community engagement and socio-economic development”. On the other hand, “coordination via institution,” although not having effect on the communication and awareness, exhibits an intensified distribution across all the other themes, particularly the “corporate governance and institutional efficiency” as determined by the color gradient. While slightly lower in magnitude than the prior, “Connectivity and Transboundary Cooperation” remains significantly impacted. It also exhibits an amplified intensity when it comes to “coordination via actors,” underscoring its vulnerability to this coordination aspect. However, “cultural heritage and historical conservation” does not seem to be affected by any coordination aspect, except coordination via institution and coordination via knowledge.

## Discussion

This discussion interprets and contextualizes the findings by addressing the three research questions, evaluating their practical implications, and relevance to policy, management, and the TFCA discourse.

### Moderate Alignment between National Park Policies

Our study revealed that policies between the two national parks show a moderate alignment (3.6 out of 5) as certain aspects of the objectives show commonality and similar implementation activities to some extent. This is to be expected given that national parks globally are established with a similar mandate, hence common objectives despite the difference in detail, priority, and scope (Gissibl et al. 2022). While moderate alignment in these policies is evident, the root causes of the discrepancy are hinged on subtle emanations of power asymmetry between the two countries. Among the three GLTFCA member states, South Africa has historically played a dominant role in agenda-setting, largely due to its greater institutional capacity, financial resources, and regional geopolitical influence. The initial impetus for the GLTP—including the convening of early meetings, the drafting of foundational policy documents, and the coordination of transboundary initiatives—was predominantly led by South African institutions. In contrast, less-resourced partners such as Zimbabwe and Mozambique were positioned more as policy adopters than co-designers, reflecting asymmetrical participation in the early stages of the TFCA’s institutional development. While this study does not empirically investigate power relations, these patterns are consistent with the broader governance scholarship, where unequal resource distribution and institutional capacity often shape participation and influence in transboundary initiatives (Büscher 2013; Duffy 2006). Hence, KNP emphasizes a comprehensive, multi-tiered approach reflecting its stronger institutional capacity and broader strategic ambition. This expansion in policy regime extent, covering complex interdependent aligns with what Bolognesi et al. (2021) conceptualized as Institutional Complexity Trap (ICT). While this enables holistic coverage increasing extent, it also risks reduced internal coherence if coordination mechanisms do not evolve proportionally, resulting in a complexity trap. The numerous guidelines and institutional mapping risks, slowing down decision-making and mounting social costs without producing offsetting benefits. This, in turn makes the system potentially less responsive to immediate environmental challenges. Maor (2020) and Holstead et al. (2021) underscored economic reparations and bureaucratic inefficiencies associated with policy over-design and complexity. In contrast, GNP follows a more hands-on, action-oriented approach, a locally grounded implementation style emphasizing community-level engagement. This means that the objectives are easily unpackaged, facilitating rapid responsiveness with each objective directly tied to tangible actions, like e.g. surveying disputed areas, HWC. However, lack of depth in institutional engagement, particularly in long-term frameworks and cooperative governance, results in oversight structures, making it difficult to track progress or hold actors accountable (Howlett and Mukherjee 2017). This divergence from KNP’s more structured, policy-heavy approach reflects a major difference in how each park navigates legal pluralism. While both countries operate under plural legal systems, Zimbabwe relatively accommodates customary law within conservation governance. In South Africa, however, state law overwhelms customary systems, which have been, to a greater extent, historically marginalized within conservation. This asymmetry in the uptake and integration of customary law creates underlying misalignments in governance logic, authority structures, and stakeholder engagement strategies between the two parks, hence the policy incoherence.

### International Frameworks and Institutional Weaknesses as Drivers of Coherence Levels

Our analysis from policy coherence shows strong alignment “Conservation and Environmental Management”; “Land Use, Regional Planning, and Resource Management”, and “Community Engagement and Socio-Economic Development”. National parks globally are founded on common overarching objectives (Gissibl et al. 2022). Moreover, these themes are inherently interconnected, often guided by structured international frameworks such as the Convention on Biological Diversity (CBD), the UNESCO Man and Biosphere Program, and the IUCN (International Union for Conservation of Nature) guidelines (Dudley 2008; Vasilijević and Pezold 2011; Andersen 2018). Hence, policy standardization is often driven by these frameworks, making conservation goals highly aligned between both parks. Overall, this suggests that normative (non-regulatory) themes, which are shaped by trends of global norms such as community engagement and strategic dimensions, which are often shaped by shared international discourses or donor frameworks (top-aligned themes), are more susceptible to policy alignment. However, alignment for “Community Engagement and Socio-Economic Development” in policy coherence doesn’t resonate the same in functional coherence. Written policy activities and actual practice present a diverging phenomenon. This has been extensively documented in the policy debates as attributed to factors such as policy ambiguity and contextual factors in policy implementation (Howlett and Rayner 2005; Fowler 2021, 2023). However, the growing emphasis on integrating community values into conservation practices has resulted in significant shifts in conservation paradigms (Mace 2014; IPBES 2021; Mpofu et al. 2025), which likely accounts for the high coherence in “Community Engagement and Socio-Economic Development”. Organizations such as the Intergovernmental Science-Policy Platform on Biodiversity and Ecosystem Services (IPBES) have championed approaches that prioritize community well-being and cultural integration, prompting changes in the policy objectives in protected areas (IPBES 2021; Munthali et al. 2023). While contemporary conservation practice reflects efforts toward decolonialization, power asymmetries in sub-Saharan Africa continue to shape institutional outcomes (Sibanda 2015; Eshuis and Gerrits 2021). In sub-Saharan Africa, colonial-era laws institutionalized through state systems coexist unequally with indigenous norms (Rautenbach 2010; Ubink et al. 2021). This top-down legal pluralism compels local systems to adapt to dominant legal frameworks, resulting in regulatory contradictions, as parks enforce formal conservation laws while communities rely on traditional norms. This aligns not only with GLTFCA but with various TFCAs similar challenges, operating under distinct yet intersecting legal regimes (Linell et al. 2019; Retief et al. 2023).

Policies from both parks highlight the need for proactive community engagement, demonstrating a shared commitment to integrate local perspectives despite variations in implementation across different locations. While policy documents reflect a stronger alignment on these issues, several scholars seem to contradict this notion as they highlight the neglection of communities (Büscher 2013; Chibememe et al. 2014; Zanamwe et al. 2018). Communities have voiced their concerns regarding the lack of awareness and inadequate information dissemination, which remains unresolved (Dhliwayo et al. 2023; Mpofu et al. 2023; Thakholi et al. 2024). Perhaps the starting point of community exclusion is this absence of landscape-related information and knowledge at their disposal, an assertion noted by various scholars (Hosseininia et al. 2016; Chiutsi and Saarinen 2017, 2019). This is further substantiated by the policy results, which indicate that “communication and Awareness” is mostly lagging when it comes to policy coherence. The absence of a communication framework cripples the functionality of TFCAs. Chirozva (2015) and Chiutsi and Saarinen (2017) have also highlighted the lack of a robust and formalized communication framework within the GLTFCA.

The absence of structured communication mechanisms contributes to confusion, exacerbating role ambiguity and creating mandate overlaps, a phenomenon associated with institutional inefficiency (Nilsson et al. 2012; Kalaba et al. 2014). This corresponds with our results in functional coherence, which show the dominance of “Corporate Governance and Institutional Efficiency” as a major barrier to coherence and coordination. Comparable challenges have also been reported in similar transboundary protected areas, particularly TFCAs, where the comprehensive framework for policy harmonization remains an ongoing priority (Büscher and Schoon 2009; Chitakira et al. 2022; Retief et al. 2023). Bureaucratic hurdles and issues of sovereignty hinder transboundary collaborations; hence, national governments and central institutions are often seen as ineffective in comprehensively facilitating coherence (Spierenburg and Wels 2006; Dressler and Büscher 2008). Literature suggests decentralization as a mechanism to ease this inefficiency as it allows management to occur incrementally, addressing distinct issues in smaller, more manageable units rather than imposing a top-down, “one-size-fits-all” solution (Ribot 2003; Murphree 2009; Clement 2010; Thaler et al. 2017). While this is not as easy as it sounds, institutional structures first have to be well coordinated horizontally and vertically, mimicking a synergistic but not fragmented model with clear roles and responsibilities, along with communication guidelines and protocols. Additionally, in the context of institutional inefficiency, cognitive factors should be taken into consideration as LG is inherently linked to uncertain human behavior, given that institutions are cognitive, normative, and regulative structures (Gilardi and Radaelli 2012; Chaffin and Gunderson 2016).

While the essence of LG is seamless cooperation, this has been met with considerable friction when it comes to TFCAs (Sjöstedt and Linell 2021; Thakholi et al. 2024). “Connectivity and Transboundary Cooperation” also remain significant challenges both in theory and practice as indicated by functional coherency results. Linell et al. (2019) highlighted the existence of TFCAs with many theoretical puzzles, further expressing that it is difficult to implement the practice without comprehending the theory. However, some scholars argue that practice precedes theory, aligning with our assertion that policies should be informed by practical challenges. The dysfunctionality of the Sengwe-Tshipise corridor which should facilitate connectivity, practically segregates GNP from other national parks. This is mainly due to bureaucratic and institutional inefficiencies which further cripples the collaboration. While the corridor might be functional for wildlife, the contemporary conservation paradigm also takes into consideration humans, a principle highlighted in the IPBES and a primary objective for the TFCAs (Wolmer 2003; Mace 2014; IPBES 2021). Therefore, concerted efforts must be made to address infrastructure gaps and improve connectivity. This can ease joint operations such as anti-poaching patrols, fire management, and habitat restoration while increasing revenue for GLTFCA member countries.

### Coordination via Knowledge and Institution as Major Barriers for Coherence

**Fig. 2:**
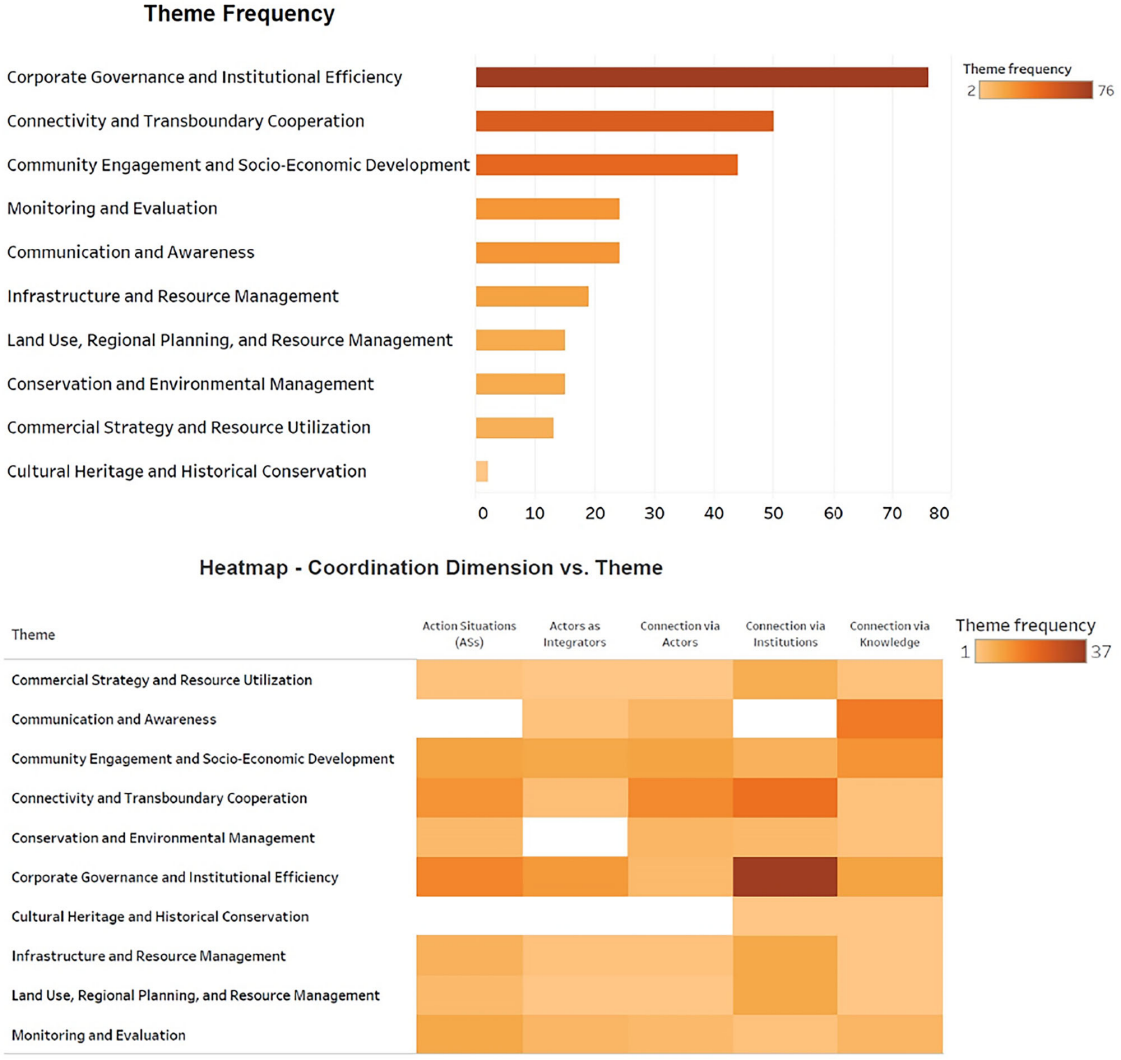
Policy theme frequency and coordination dimensions in transboundary conservation governance. (Top): Bar graph displaying the frequency of ten policy themes across Kruger and Gonarezhou National Parks, highlighting dominant focus areas in strategic documents. (Bottom): Heatmap illustrating the interaction between each policy theme and five coordination dimensions based on (Dombrowsky et al. 2022) conceptual framework on coordination aspects

Given that coordination aspects serve as key dimensions that determine how well different institutions and actors work together to achieve common goals, our results reveal major flaws in the “coordination via knowledge” aspect. This also corresponds to the policy analysis results, which indicated shortcomings in “communication and awareness”. While communication precedes knowledge, these two are interrelated (Tengö et al. 2014; Galafassi et al. 2018). A detailed analysis further indicated that within functional coherence, “Community Engagement and Socio-Economic Development” suffers the most impact under “Coordination via knowledge”. This can be attributed to either fragmented information sharing, lack of access to critical information or unidirectional knowledge flow. Without communication, knowledge co-creation is impossible hence, information remains the backbone of all processes going forward (Sayer et al. 2013; Tengö et al. 2014). Transdisciplinary research has advanced, recognizing that knowledge is non-linear, multi-dimensional, and co-created (Schneider et al. 2019; Gugerell et al. 2023). However, many conservation areas still rely primarily on scientific and technocratic knowledge (Ainsworth et al. 2020; Torrents-Ticó et al. 2021; Jessen et al. 2022). While models for weaving knowledge exist, conservationists should leverage these approaches to address challenges from a multi-dimensional knowledge perspective. This also explains why coherence challenges are more prominent at the process level than the outcome level (Fig. 2), as the process level is mainly facilitated through effective communication before anything can be put to the next stage (Dombrowsky et al. 2022). Although communication is two-way, the challenges associated with the transfer of tacit knowledge are well-acknowledged (Connell et al. 2003; Schmidt 2012). However, a structured and clear model for transdisciplinary knowledge sharing and information exchange remains absent in the GLTFCA. In this contemporary era, often referred to as the “Information Age”, technological advancements have significantly transformed knowledge dissemination (Goldman and Scardamalia 2013; Soma et al. 2016). In previous decades, perhaps we would have argued strongly on limited technological advancement as a barrier to knowledge, awareness, and information. However, the perpetuation of communication and knowledge co-creation challenges today points to factors beyond technological access. Such challenges can only be attributed to institutional inefficiency, which is also echoed in our results for both policy and functional coherence analysis.

Our results also show that “Coordination via institution” aspect presents another significant challenge with ties to “Corporate Governance and Institutional Efficiency”. Despite previous studies attributing these challenges to resource limitations (Chiutsi and Saarinen 2017; Kansky 2022), which to some extent is true, it should not be regarded as the primary factor, lest one succumb to a scarcity mindset. The concept of adaptive governance even highlights the importance of flexibility, innovation, and the ability to navigate constraints (Folke et al. 2005; Yasmin et al. 2020). However, this approach must be complemented by the notion of a minimum resource threshold, which asserts that a certain baseline of resources is essential for the effective functioning of governance systems (Park et al. 2024). Unproductive meetings have been noted as a major setback, as several respondents indicated that these meetings fail to produce actionable outcomes despite their frequency. This situation has contributed to policy alienation a condition in which actors experience diminishing engagement and effectiveness due to continuous policy implementation challenges (Tummers et al. 2015; van Engen et al. 2016). This further diminishes individual morale and motivation, leading to cascading inefficiencies that extend from lower levels of the organization to leadership, a phenomenon conceptualized as “illusion of participation” (Forde 2005; Dawodu et al. 2021). This illusion is also attributed to the complicated instructional structures to which participants alluded to. There is no doubt that GLTFCA has unintentionally established of a pattern of delayed policy implementation as reiterated by respondents (Bhatasara et al. 2013; Retief et al. 2023; Thakholi et al. 2024). This reinforces institutional inefficiency, often resulting in erosion of trust, a foundational element in governance and institutional stability, particularly from ground-level actors (Sayer et al. 2013; Westerink et al. 2017).

## Conclusion

In conclusion, this study assessed policy coherence between Kruger and Gonarezhou National Parks within the GLTFCA context. Policy coherence results reveal a moderate alignment (3.6 out of 5) within the strategic objectives of the two parks, reflecting a shared overarching vision for conservation and development. However, this alignment remains partial, with the moderate score pointing to underlying challenges such as power asymmetries, divergent implementation approaches, and context-specific institutional dynamics that hinder full coherence. Strong alignment was observed in policy areas that are mainly influenced by international frameworks (e.g., IUCN, SDGs, CBD), which are likely driven by donor priorities and global norms.

In contrast, themes related to governance, institutional efficiency, and transboundary cooperation showed the weakest coherence, highlighting implementation gaps and fragmented accountability. These weaknesses correspond closely to key coordination dimensions, particularly those related to institutional alignment and knowledge-sharing mechanisms. These findings underscore that while policy intent aligns, functional coherence is constrained by disparities in institutional capacity, complexities of legal pluralism, and fragmented coordination mechanisms. These constraints reveal critical areas for targeted improvement in TFCAs. Considering that TFCAs are grappling with ongoing coordination and collaboration challenges, this study provides empirical insights into the current disconnect between policy provisions and operational realities, highlighting critical areas in need of immediate attention and resource allocation. Recognizing and addressing on-the-ground challenges is imperative for shaping effective policies, ensuring conservation success, and meeting global environmental targets as part of international agreements such as the IUCN, CBD, and SDGs, to which South Africa and Zimbabwe are signatories. While various dimensions of coordination exist, they are often overlooked in research. The assessment of coordination provides a deeper understanding of where coordination breakdowns occur, resulting in targeted actions to address them (Pindyck 2013; van Beek et al. 2020). This research also contributes to ongoing efforts for policy harmonization within the GLTFCA by advancing the conceptual framework for policy and functional coherence assessment. Furthermore, the study contributes to broader debates on TFCA and landscape governance, providing a vital perspective on the interplay between policy and practice in TFCAs. Further research that comprehensively analyses policies across all three GLTFCA countries is necessary to ensure overall policy and functional coherence within the GLTFCA.

## Supplementary information

Appendix 1

Appendix 2

Appendix 3

Supplementary information

## Data Availability

No datasets were generated or analysed during the current study.
