# Supplementary material for: Fragmented Governance, Shared Landscapes: Policy and Functional (In)Coherence Insights from the Great Limpopo Transfrontier Conservation Area
Source: Environ Manage. 2025 Nov 17;76(1):1. doi: 10.1007/s00267-025-02309-9 (PMC12628470; doi:10.1007/s00267-025-02309-9)
Supplement: Supplementary file 1 — Appendix 1 [file 267_2025_2309_MOESM1_ESM.docx]

Appendix 1

Table 4: Fundamental Policies for the KNP and the GNP i.e The Park Management Plans

| **Policies Title** | **Download Link** |
| --- | --- |
| Gonarezhou Conservation Trust: Strategic Plan 2019-2023 | Policy to be provided on request. No download link available |
| Kruger National Park: Park Management Plan 2018 - 2028 | <https://www.sanparks.org/wp-content/uploads/2021/06/knp-approved-plan.pdf> |
