# Supplementary material for: Fragmented Governance, Shared Landscapes: Policy and Functional (In)Coherence Insights from the Great Limpopo Transfrontier Conservation Area
Source: Environ Manage. 2025 Nov 17;76(1):1. doi: 10.1007/s00267-025-02309-9 (PMC12628470; doi:10.1007/s00267-025-02309-9)
Supplement: Supplementary file 2 — Appendix 2 [file 267_2025_2309_MOESM2_ESM.docx]

Appendix 2

Table 5: Definitions and explanations of terms used in the analytical framework

| **Definition** | **Example in the GLTFCA Context** |
| --- | --- |
| **Policy coherence:** The alignment of policies with regards to their objectives, implementation mechanisms, and overall scope. | A wildlife conservation policy in one country allowing sustainable hunting, while another strictly prohibits it, leading to conflicts in cross-border wildlife management. |
| **Functional coherence:** consistency in the distribution of governance functions within the governing body. | Responsibilities for anti-poaching operations are clearly defined, ensuring that law enforcement across Kruger, Gonarezhou, and Limpopo National Parks is well-coordinated without duplication or gaps. |
| **Government functions:** The roles and responsibilities undertaken by governing bodies to regulate, manage, and support conservation efforts. (law development, knowledge co-creation, coordination, law enforcement, planning, conflict resolution and policy implementation) | The development and enforcement of cross-border environmental regulations, such as standardized procedures for wildlife translocation and anti-poaching protocols within the GLTFCA |
| **Coordination at process level:** The execution of governance strategies that facilitate the integration of the interests of relevant actors within the decision-making process. | Regular cross-border meetings between conservation officials from South Africa, Zimbabwe, and Mozambique to discuss shared wildlife crime challenges and coordinate responses. |
| **Coordination at outcome level**: Collaboratively developed strategies, policies, or plans incorporating the interests of key actors. | A transboundary fire management strategy developed jointly by Kruger, Gonarezhou, and Limpopo National Parks to address seasonal wildfires collaboratively. |
| **Aspects of coordination:** Different ways in which organizations or actors interact and align their efforts to achieve common goals. | Connection via institutions, connection via knowledge aspect, connection via actors, actors as integrators |
