# Supplementary material for: Fragmented Governance, Shared Landscapes: Policy and Functional (In)Coherence Insights from the Great Limpopo Transfrontier Conservation Area
Source: Environ Manage. 2025 Nov 17;76(1):1. doi: 10.1007/s00267-025-02309-9 (PMC12628470; doi:10.1007/s00267-025-02309-9)
Supplement: Supplementary file 3 — Appendix 3 [file 267_2025_2309_MOESM3_ESM.docx]

Appendix 3

Table 6: Descriptions and explanations of connection aspect/aspects of coordination.

| **Aspect of Coordination** | **Meaning** | **Explanation** |
| --- | --- | --- |
| Connection via Institutions | Refers to the formal and informal institutions that facilitate coordination between organizations (e.g., laws, policies, agreements). | Institutions set the rules of engagement, creating a framework for organizations to align their actions, ensuring that their goals and procedures are consistent and cooperative. |
| Connection via Knowledge | Emphasizes the importance of sharing information and knowledge among organizations or actors involved. | Knowledge exchange enables mutual understanding, helping organizations make informed decisions and solve problems collaboratively. |
| Connection via Actors | Refers to the individuals or organizations that are involved in coordinating efforts, including government agencies, NGOs, and other stakeholders. | The effectiveness of coordination depends on how well the individuals or organizations involved communicate, collaborate, and align their actions to meet common goals. |
| Actors as Integrators | Some actors play the role of integrators, ensuring alignment and harmonization of activities across various organizations and sectors. | Integrators act as intermediaries, bridging gaps between organizations and sectors, ensuring coordination, alignment of goals, and that efforts are not duplicated. |
| Coordination via Action Situations (AS) | Refers to the degree of coordination achieved through specific governance functions implemented across sectors. It assesses the integration and centrality of these functions within a horizontal network of actions. | This aspect measures the impact and degree of centrality of coordination across sectors. A higher score indicates that coordination instruments are implemented and have a significant impact, while a low score suggests poor connection and limited impact within the governance network. |
