# Supplementary material for: Fragmented Governance, Shared Landscapes: Policy and Functional (In)Coherence Insights from the Great Limpopo Transfrontier Conservation Area
Source: Environ Manage. 2025 Nov 17;76(1):1. doi: 10.1007/s00267-025-02309-9 (PMC12628470; doi:10.1007/s00267-025-02309-9)
Supplement: Supplementary file 4 — Supplementary information [file 267_2025_2309_MOESM4_ESM.docx]

Table 7: Comparative analysis of policy objectives between Kruger National Park (KNP) and Gonarezhou National Park (GNP), highlighting their alignment. The table presents both objective-level and theme-based alignment scores (ranging from 1 to 5), accompanied by justifications for each score.

| **Theme** | **Objective (KNP - South Africa)** | **Objective (GNP - Zimbabwe)** | **Comment** | **Alignment Score (OB)** | **Alignment Score (theme)** |
| --- | --- | --- | --- | --- | --- |
| Connectivity and Transboundary Cooperation | To improve transboundary disease management within the wildlife-livestock-human interface through enabling institutional coordination and partnerships. | No direct match. | KNP focuses on disease management and coordination, while GNP does not explicitly address transboundary disease concerns. | 0 | 4.1 |
|  | To ensure the implementation of the GLTFCA improved land and air access program. | Lobby for & support improved border services & improved air & road access & routes. | Both parks prioritize improving transboundary accessibility, but GNP explicitly mentions border services and access improvements. | 5 |  |
|  | To support and enhance the implementation of the GLTFCA conservation and cultural management program. | Conduct consultations regarding cultural sites; commission historian to document & describe cultural sites & practices. | GNP focuses more on cultural heritage documentation rather than broader conservation and cultural management programs. | 3 |  |
|  | To support the implementation of joint responsible tourism, marketing, and branding programs and activities. | Support development of cross-border tourism events, products & circuits to promote tourism. | Both parks aim to strengthen cross-border tourism efforts, though GNP has a more tourism-centric approach. | 5 |  |
|  | To support the implementation of joint socio-economic programs and activities (GLTFCA). | Resilient livelihoods development, gender equality & reproductive health, sport & cultural initiatives. | Both align on socio-economic development, though GNP places more emphasis on local community initiatives. | 4 |  |
|  | To support the implementation of a joint safety and security program. | Cooperate with buffer area managers in law enforcement; work with ZRP & judiciary to improve prosecution outcomes. | Both parks focus on security, but GNP emphasizes law enforcement partnerships while KNP includes broader security programs. | 5 |  |
|  | To develop stakeholder and communication plans. | Mphuka: AGM & Sector Meetings; Mohuka advisory committee, park visits; problem solving, liaison & advisory. | Both prioritize stakeholder engagement, though GNP focuses on advisory committees rather than formal communication plans. | 4 |  |
|  | To develop and support the implementation of joint capacity development, awareness, training, and research programs. | Identify & action priority research projects & establish research centre; develop GNP facilities & awareness for tourists & other target groups. | GNP emphasizes research and awareness-building, while KNP includes broader training and capacity development. | 4 |  |
|  | To guide peripheral conservation land use practices in support of compatible environmental and socio-economic outcomes. | Build relations with buffer area managers & stakeholders; support land use planning in buffer areas & subsequent implementation. | Both align on guiding land-use planning and buffer zone conservation. | 5 |  |
|  | To ensure the boundary descriptions and land audit register reflect accurate information. | Survey all disputed areas; discuss surveys with stakeholders, create awareness of location & resolve any disputes; demarcate boundaries. | Both parks focus on boundary accuracy and land audit processes. | 5 |  |
|  | To monitor and evaluate the impact of the implementation programs and adapt as required. | Develop & implement monitoring, evaluation & learning systems. | Both parks aim for ongoing evaluation and adaptive management. | 5 |  |
| Commercial Strategy and Resource Utilization | To ensure and improve financial sustainability. | Identify key donors and nurture relationships. | Both focus on financial sustainability and donor relations, but KNP has a broader objective regarding park-wide sustainability. | 4 | 3.8 |
|  | To attain effective financial management. | Maintain up-to-date and accurate financial records and systems. | Both aim for effective financial management systems, with GNP emphasizing record-keeping and systems. | 4 |  |
|  | To improve the park’s image amongst its stakeholders through the provision of well-planned, managed, and coordinated events. | Develop & implement a strategic marketing plan. | Both parks engage with stakeholders through organized events, but KNP focuses more on event planning for image improvement. | 3 |  |
|  | To support the implementation of joint responsible tourism, marketing, and branding programmes and activities. | Support development of cross-border tourism events, products & circuits to promote tourism. | Both KNP and GNP prioritize joint tourism development, with GNP emphasizing cross-border initiatives. | 3 |  |
|  | To grow revenue (Including alternative sources of revenue). | Support development of cross-border tourism events, products & circuits to promote tourism. | Both focus on growing revenue through tourism and strategic marketing, but KNP’s approach is broader and more business-oriented. | 3 |  |
|  | To leverage strategic business partnerships, and participate in economic clusters and planning processes. | Engage service providers for compelling, authentic content; Attend trade shows & develop relationships with key DMC’s. | Both aim to foster partnerships and collaborate with service providers, but KNP focuses more on economic clusters and business models. | 4 |  |
|  | To facilitate tourism access opportunities. | Support development of cross-border tourism events, products & circuits to promote tourism. | Both emphasize tourism access and cross-border initiatives but GNP emphasizes specific border access and tourism event facilitation. | 4 |  |
|  | Ensure optimal returns from commercial operations. | Gonarezhou branded merchandise; Value-added services, e.g., game drives, walks, vehicle hire. | Both focus on commercial operations and value-added services, but KNP does not have a direct match for merchandise development. | 4 |  |
|  | To market the park tourism products, facilities, and activities. | Develop Park-run tourism options that broaden target market & increase price point. | Both aim to expand and market tourism, but GNP focuses more on developing park-run options for a broader market. | 5 |  |
| Corporate Governance and Institutional Efficiency | To provide support to SANParks, whilst strengthening enabling institutional partnerships. | Build and strengthen stakeholder relationships supportive of the park’s mandate. | Both prioritize institutional partnerships, with KNP supporting SANParks and GNP focusing on broader stakeholder relationships. | 5 | 3.4 |
|  | To strengthen good co-operative arrangements with respect to DCA management. | No direct match. | KNP focuses specifically on DCA management, while GNP does not emphasize this as a core objective. | 0 |  |
|  | To create and maintain a sound working environment through fair and equal treatment of all employees and stakeholders to deliver SANParks strategic objectives. | Review and improve HRPP; Staff performance management. | Both prioritize fair treatment and human resources management, but KNP emphasizes SANParks' strategic alignment, while GNP focuses on staff development. | 5 |  |
|  | To attract and grow science capacity, skills, and human capital. | Clarify ecological & threat monitoring (ETM) priorities; Identify & action priority research projects & establish research centre. | Both KNP and GNP emphasize the growth of science and research capacity, though KNP has a broader strategic focus. | 4 |  |
|  | To ensure the park attracts and retains the most suitable human capital. | Continually review staff structures vs. needs; Staff performance management. | Both focus on human capital retention, with KNP more focused on strategic HR alignment and GNP on performance management. | 5 |  |
|  | To implement plans and skills development strategies to meet the strategic goals of the organization. | Build capacity and develop staff. | Both focus on staff development, but GNP is more focused on direct capacity building. | 5 |  |
|  | To manage and reduce the impacts of park activities in accordance with legislation to prevent pollution and environmental degradation. | No direct match. | KNP focuses on legal compliance and environmental management, while GNP has no direct mention of this in its objectives. | 0 |  |
|  | To strengthen collaboration and partnerships at the strategic level. | Build relations with buffer area managers & stakeholders. | Both aim to strengthen partnerships, but GNP focuses more on specific buffer zone relations. | 4 |  |
|  | To review institutional arrangements to harmonize alignment and leverage enabling partnerships. | Participate in JPMC & other GL-TFCA initiatives & structures. | Both emphasize institutional arrangements, but GNP focuses more on transboundary collaboration (GL-TFCA). | 4 |  |
|  | To build and strengthen stakeholder relationships supportive of the park’s mandate. | Mphuka: AGM & Sector Meetings; Mohuka advisory committee, park visits. | Both parks prioritize stakeholder relationships, though KNP focuses on broad image-building and coordination, while GNP emphasizes direct community engagement. | 5 |  |
|  | To support stewardship/conservation and offset priorities through cooperative partnerships. | No direct match. | KNP’s stewardship focuses on long-term ecological conservation, which is not explicitly mentioned in GNP’s objectives. | 0 |  |
| Cultural Heritage and Historical Conservation | To support the implementation of the GLTFCA conservation and cultural management programme. | Participate in JPMC & other GL-TFCA initiatives & structures | Both KNP and GNP share a focus on the GLTFCA initiatives, emphasizing the integration of cultural heritage in the regional management frameworks. | 5 | 3.8 |
|  | To develop new CH tourism products. | Develop Park run tourism options that broaden target market & increase price point | Both objectives focus on tourism development, but KNP specifically targets cultural heritage (CH), while GNP is more broadly focused on tourism expansion. | 4 |  |
|  | To facilitate the second phase of excavation at Thulamela. | Conduct consultations re. cultural sites | KNP's focus on excavations aligns with GNP's consultation and site assessments, though the activities differ in nature. | 3 |  |
|  | To improve the understanding of the CH landscape of the park. | Assess status & feasibility of cultural sites | Both objectives aim at understanding and assessing the cultural landscape, but KNP's goal is broader, while GNP focuses on the feasibility and documentation of specific sites. | 4 |  |
|  | To build staff awareness of CH. | Support conservation education & Park visits for local schools | KNP aims to raise staff awareness, while GNP focuses on broader community awareness and conservation education. | 3 |  |
|  | To manage CH sites and collections in accordance with legislation and minimum standards. | Ensure GCT staff understand key conservation & sustainability principles & the importance of GNP in landscape | KNP focuses on CH-specific legislation and standards, while GNP emphasizes general conservation and sustainability principles, but they overlap in the staff capacity building aspect. | 4 |  |
| Monitoring and Evaluation | To monitor and evaluate the impact of the implementation programmes and adapt as required. | Implement improved planning systems, reporting systems, and monitoring, evaluation, and learning systems | Both KNP and GNP focus on monitoring and evaluation, with KNP more program-focused and GNP on system improvements and implementation frameworks. | 4 | 3.3 |
|  | To maintain and refine decision support systems for river management, integrating monitoring data, desired states, and TPCs to monitor and evaluate reserve implementation. | Clarify ecological & threat monitoring (ETM) priorities, collect baseline & monitoring data | KNP’s river management is more specialized, while GNP’s approach to ecological monitoring is broader. Both focus on integrating data for management. | 4 |  |
|  | To develop and manage an effective system to store, share, and report on data, contributing to learning and preserving institutional memory. | Develop and implement monitoring, evaluation, and learning systems | Both KNP and GNP focus on developing systems for data collection, management, and learning, although GNP’s focus is more on the technical implementation of systems. | 4 |  |
|  | To conduct and facilitate relevant and robust socio-ecological research and monitoring to support the core pillars of SANParks. | Support & training in mitigation techniques | KNP focuses on socio-ecological research, while GNP’s focus on mitigation techniques for specific threats is a more targeted approach. | 3 |  |
|  | To ensure science supports management actions, underpins decision-making, and contributes towards effective park management and policy development. | Work with partners to increase communities' awareness of CBNRM | KNP’s focus on science-driven decision-making aligns with GNP’s community-driven awareness efforts, though GNP is more specific to CBNRM (Community-Based Natural Resource Management). | 3 |  |
|  | To ensure monitoring and evaluation of programme implementation and effectiveness. | Develop and contribute to a robust disease surveillance system to detect unusual/epidemic disease events | While KNP’s monitoring is broad, GNP’s disease surveillance system is a specialized type of monitoring. | 3 |  |
|  | To effectively contribute to the Human-Wildlife Conflict (HWC) knowledge base and applied research. | System to respond, assess & react to cases of HWC | Both objectives focus on addressing human-wildlife conflict, with KNP aiming for research contributions, and GNP focusing on actionable systems and responses. | 4 |  |
|  | To monitor and evaluate the impact of human-wildlife conflict interventions on environmental, social, economic, and institutional/legislative outcomes. | System to respond, assess & react to cases of HWC | Both KNP and GNP focus on the impact of human-wildlife conflict but in different manners; KNP focuses more on comprehensive evaluation, while GNP is more about responding to immediate cases. | 4 |  |
|  | To develop an understanding of the human-wildlife interface to better inform, support, and monitor responsible land use management practices. | Build relations with buffer area managers & stakeholders | While KNP focuses on the broader human-wildlife interface, GNP’s focus is more on collaborative relationships with buffer area managers, which is part of land-use management. | 4 |  |
|  | To review, amend, and provide inputs into the internal policy framework, external institutional arrangements, and the National and International legislative framework. | Provide input to reformed hunting regulations at local & national level | KNP focuses on a broad review of policies, while GNP specifically addresses hunting regulations. | 3 |  |
|  | To monitor, evaluate, and review the Communication Programme. | Survey all disputed areas | Both focus on evaluations, but KNP looks at communication, while GNP evaluates land use and boundary-related issues. | 2 |  |
|  | To develop and implement a records management and file plan for the park in accordance with SANParks policies and procedures. | Implement improved planning systems | KNP focuses on records management, while GNP’s approach is more related to improving overall planning systems, which may include records management. | 3 |  |
|  | To ensure proper inventory management. | Conduct hunt monitoring | KNP focuses on inventory management, while GNP is more focused on specific wildlife management through hunting monitoring. | 2 |  |
|  | To develop and contribute to a robust disease surveillance system to detect unusual/epidemic disease events. | Support & training in mitigation techniques | KNP’s disease surveillance system focuses on health-related threats, while GNP focuses on specific conflict mitigation techniques. | 3 |  |
|  | To evaluate and respond to disease threats to wildlife posed by humans and domestic livestock and vice versa. | Support review of relevant national policies & regulatory frameworks | KNP focuses on disease evaluation and response, whereas GNP looks at policy frameworks related to regulations. | 3 |  |
|  | To ensure fire monitoring and research. | Develop & implement fire management plan | Both KNP and GNP emphasize fire management, but KNP focuses more on research and monitoring, while GNP prioritizes planning and execution. | 4 |  |
|  | To monitor short-term responses and long-term changes in the groundwater table level in response to climatic cycles. | Identify & action priority research projects & establish research centre | KNP’s focus on groundwater levels is more specific, while GNP’s focus is on establishing research priorities that could include water management. | 3 |  |
|  | To inventorize and characterize non-riverine aquatic systems (e.g., wetlands, pans, springs). | Clarify ecological & threat monitoring priorities | KNP focuses on specific aquatic systems, while GNP’s monitoring is more general across various ecological threats. | 3 |  |
|  | To evaluate potential risks, source areas, and pathways of invasion into the park at a regional scale. | Assess & mitigate invasive spp. threats | Both objectives address invasive species, but KNP’s focus is on evaluation at a regional scale, while GNP is more focused on direct mitigation. | 4 |  |
| Communication and Awareness | To effectively communicate socio-ecological science in order to create awareness, educate, and build constituency with a broad base of academic and non-academic stakeholders. | Support conservation education & Park visits for local schools. | Both aim to build awareness and education on conservation. KNP focuses on a broader academic and non-academic base, while GNP focuses more on local schools and communities. Justification: The difference in target audiences justifies a moderate match. | 4 | 2.8 |
|  | To develop a communication plan as informed by the internal and external Stakeholder Engagement Plan. | Create & strengthen community-park partnerships in tourism development. | KNP is focused on broader communication planning, while GNP emphasizes community partnerships in tourism. Justification: The alignment is moderate because both emphasize stakeholder engagement, but the approaches differ. | 4 |  |
|  | To inform the public through mass media about major developments or incidents in the park. | Drive a coordinated positive social media campaign through multiple channels. | Both focus on informing the public, but KNP uses mass media while GNP focuses more on digital/social media platforms for outreach. Justification: There is alignment in the communication goal but differences in the medium used. | 4 |  |
|  | To govern the implementation and support of outreach action projects and awareness according to a framework and clear guidelines. | Develop & implement a strategic marketing plan. | KNP focuses on outreach programs with a governance framework, while GNP focuses on marketing strategies for tourism. Justification: The focus on marketing in GNP vs. governance in KNP results in a moderate match. | 2 |  |
|  | To create conservation awareness within the staff of the KNP. | Ensure GCT staff understand key conservation & sustainability principles & the importance of GNP in landscape. | Both focus on staff awareness regarding conservation, but KNP’s approach is broader across various levels, while GNP focuses on the staff of the GCT. Justification: There is conceptual alignment, but the specifics differ regarding staff focus. | 4 |  |
|  | To develop and implement a comprehensive environmental education and social legacy program to address the needs of multi-stakeholder groups. | Support schemes which allow legal & sustainable use of Park resources. | KNP's focus on environmental education is more holistic and programmatic, whereas GNP focuses on legal and sustainable resource use. Justification: Both are addressing conservation, but GNP is more focused on sustainable resource use, thus the match is partial. | 3 |  |
|  | To promote an appreciation of wilderness amongst the general public, especially visitors to the park. | Develop Park run tourism options that broaden target market & increase price point. | Both aim to promote the park, but KNP focuses on wilderness appreciation, while GNP emphasizes tourism development and increasing market engagement. Justification: The focus is on promotion, but one is more about wilderness and the other about tourism expansion. | 4 |  |
|  | To facilitate a speedy flow of information between park management and staff using bulletins and internal newsletters, as well as to respond to general customer queries. | No direct match | This is a misalignment, as KNP is more focused on internal communication, while GNP’s objective deals with infrastructure maintenance. Justification: The difference in focus on communication systems vs. infrastructure maintenance results in a low match. | 0 |  |
|  | To ensure electrical, two-way radio communication, and mechanical equipment are serviced and maintained to acceptable standards. | Procure necessary equipment. | Both KNP and GNP are concerned with maintaining operational standards. However, KNP focuses specifically on communication systems, while GNP is more concerned with procuring equipment generally. Justification: The difference in operational focus results in a moderate match. | 3 |  |
|  | To develop stakeholder and communication plans. | No direct match | KNP is focused on stakeholder and communication planning, while GNP focuses on infrastructure development. Justification: There is conceptual alignment in planning, but the focus on infrastructure vs. communication planning results in a low match. | 0 |  |
| Infrastructure and Resource Management | To improve tourist viewing and safety. | Develop & implement fire management plan. | KNP focuses on improving the tourist experience and safety, while GNP emphasizes operational aspects such as fire management. Justification: The alignment is moderate since both aim to improve park management and safety but through different approaches. | 1 | 3 |
|  | To support stewardship/conservation and offset priorities through cooperative partnerships. | Create & strengthen community-park partnerships in tourism development. | Both emphasize partnerships, but KNP emphasizes stewardship and conservation, while GNP focuses on tourism development partnerships. Justification: The conceptual alignment is moderate, with differences in the nature of partnerships. | 4 |  |
|  | To upgrade and construct new infrastructure. | Develop & implement Infrastructure Development Plan. | Both KNP and GNP focus on infrastructure development, but KNP focuses more on upgrades and new construction, while GNP provides a broader developmental plan. Justification: There is a moderate alignment, as both focus on infrastructure, but GNP’s approach is more comprehensive. | 4 |  |
|  | To provide the same choices for all consumers to ensure the full participation of persons with disabilities, the elderly and parents with young children by creating appropriate facilities and providing dignified service. | Ensure routine maintenance of infrastructure & equipment. | KNP emphasizes inclusivity and accessibility for a diverse range of consumers, while GNP focuses on maintaining infrastructure for general use. Justification: The specific focus on inclusivity in KNP results in a moderate match, as GNP addresses general infrastructure needs. | 3 |  |
|  | Align new and existing tourism infrastructure and tourism products to be in line with market demands and industry standards to enable revenue optimisation. | Gonarezhou branded merchandise. | KNP focuses on aligning infrastructure and products with market demand, while GNP focuses on branded merchandise as part of their tourism offer. Justification: GNP’s specific emphasis on merchandise results in partial alignment compared to KNP’s broader infrastructure alignment. | 3 |  |
| Community Engagement and Socio-Economic Development | To promote sustainable local and regional economic development. | Resilient livelihoods development | Both objectives focus on economic sustainability, but KNP has a broader scope, incorporating regional economic growth, while GNP emphasizes community resilience. | 4 | 4.4 |
|  | To support the implementation of joint socio-economic programmes and activities. | Develop & implement a performance-linked benefit-sharing scheme | Both focus on socio-economic benefits, but KNP's goal is broader, while GNP is more focused on performance-based benefits. | 4 |  |
|  | To develop stakeholder and communication plans. | Mphuka: AGM & Sector Meetings; Mohuka advisory committee, park visits; Problem solving, Liaison & advisory | KNP's objective is more structured in terms of planning, while GNP focuses on direct engagement mechanisms. | 4 |  |
|  | To manage human conflict with animals inside the park and reduce impacts associated with human-wildlife interactions. | System to respond, assess & react to cases of HWC; Support & training in mitigation techniques | Both address human-wildlife conflict, but GNP includes structured response mechanisms and mitigation training. | 5 |  |
|  | To develop and implement internship, learnership, and volunteer programmes for communities and target groups. | Ensure GCT recruitment prioritizes local employment | GNP supports local employment but lacks a structured internship and volunteer initiative. | 4 |  |
|  | To identify and facilitate appropriate partnerships at various levels for community benefit-sharing. | Create & strengthen community-park partnerships in tourism development | Both objectives align in fostering community partnerships for economic benefits. | 5 |  |
|  | To facilitate and support the settlement and implementation of land claims in the park through beneficiation scheme initiatives. | Survey all disputed areas; Discuss surveys with stakeholders, create awareness & resolve disputes; Demarcate boundaries; Conduct ground-truthing field visits with stakeholders | Both address land claims, but GNP has a more structured approach to resolving disputes and demarcation. | 5 |  |
|  | To develop and implement a local enterprise support programme. | Support schemes that allow legal & sustainable use of park resources | Both objectives support local enterprise, but KNP’s approach is broader, while GNP focuses on sustainable resource use. | 4 |  |
|  | To facilitate job creation in local communities through EPWP and other infrastructure development initiatives. | Ensure GCT recruitment prioritizes local employment | Both focus on job creation, but KNP includes broader infrastructure-based initiatives. | 4 |  |
|  | To facilitate community access to and use of significant cultural sites. | Conduct consultations regarding cultural sites; Commission historian to document & describe cultural sites & practices; Assess status & feasibility of sites; Develop awareness materials around culture & history; Correct va-Tsonga place names in GNP | Both emphasize cultural preservation, but GNP has a more detailed plan with documentation, feasibility assessments, and awareness campaigns. | 5 |  |
| Land Use, Regional Planning, and Resource Management | To develop enabling institutional arrangements, sector planning, and compatible land-use management reports. | Support land-use planning in buffer areas & subsequent implementation | Both focus on land-use planning, but KNP emphasizes institutional arrangements, while GNP focuses on buffer areas. | 4 | 4.3 |
|  | To promote bioregional planning and integration through promoting co-operative partnerships with biospheres. | Identify potential/existing corridors & conduct feasibility study; Support land-use planning & subsequent implementation; Cooperate with corridor area managers in law enforcement | Both aim to integrate conservation into broader planning, but GNP focuses on corridors and enforcement. | 4 |  |
|  | To provide inputs into Bioregional Plans (BRP) and Environmental Management Frameworks (EMF). | Participate in JPMC & other GL-TFCA initiatives & structures | Both involve regional conservation planning, but KNP is more policy-oriented while GNP is more action-driven. | 4 |  |
|  | To align with and inform relevant municipal planning processes. | Support land-use planning in buffer areas & subsequent implementation | Both deal with land-use integration, but KNP focuses on municipal alignment while GNP works on buffer zones. | 4 |  |
|  | To guide peripheral conservation land use practices in support of compatible environmental and socio-economic outcomes. | Work with partners to increase communities’ awareness of CBNRM; Support efforts to improve transparency & accountability | KNP focuses on land use for conservation, while GNP supports sustainable conservation through community awareness. | 4 |  |
|  | To ensure the boundary descriptions and land audit register reflect accurate information. | Survey all disputed areas; Discuss surveys with stakeholders, create awareness & resolve disputes; Demarcate boundaries; Conduct ground-truthing field visits with stakeholders | Both focus on boundary accuracy, but GNP has a structured dispute-resolution process. | 5 |  |
|  | To expand the wilderness area. | Identify key future value areas to be retained for their wilderness value | Both aim to expand conservation areas, but GNP includes value assessments. | 5 |  |
|  | To improve protected area management effectiveness at the park and regional Greater Kruger conservation network levels. | Develop & refine effective management systems; Implement improved planning systems | Both aim to improve management but use different strategies, with GNP focusing on system improvements. | 5 |  |
| Conservation and Environmental Management | To promote responsible natural resource management and restoration of land. | Support schemes which allow legal & sustainable use of Park resources. | Both objectives emphasize sustainable resource use. KNP focuses on responsible management and land restoration, while GNP highlights legal and sustainable utilization. | 5 | 4.1 |
|  | To restore and rehabilitate processes, patterns, and viewsheds to a state of naturalness. | Assess & mitigate invasive species threats. | KNP aims for broad ecological restoration, whereas GNP focuses on addressing invasive species as a specific restoration strategy. | 4 |  |
|  | To promote the park’s riverine needs and aspirations for achieving river management goals (in terms of flows, quality, river health, etc.). | Clarify ecological & threat monitoring (ETM) priorities; collect baseline & monitoring data. | KNP emphasizes river management goals, while GNP focuses on ecological monitoring, which can include riverine ecosystems but is broader in scope. | 4 |  |
|  | To prevent (where possible) and reduce poison and pollution incidences. | Conduct hunt monitoring; ensure trophy monitoring feeds into quota setting. | KNP addresses pollution prevention directly, whereas GNP's objectives related to monitoring hunting activities may contribute indirectly to reducing environmental contaminants. | 3 |  |
|  | To understand the effects of poison and pollution on biodiversity. | Identify & action priority research projects & establish research centre. | Both parks prioritize research; KNP focuses on pollution impacts on biodiversity, while GNP aims to identify and act on various research priorities, potentially including pollution studies. | 4 |  |
|  | To support, manage, and monitor sustainable resource use through a range of co-operative arrangements. | Support schemes which allow legal & sustainable use of Park resources. | Both objectives align in promoting sustainable resource use through cooperative arrangements and legal frameworks. | 5 |  |
|  | To manage the utilization of natural and cultural resources according to ecologically sound principles, guidelines, and criteria. | Support review of relevant national policies & regulatory frameworks. | KNP focuses on ecological principles for resource utilization, while GNP emphasizes policy and regulatory reviews to support sustainable use, indicating a strategic alignment in achieving sustainable resource management. | 4 |  |
|  | To promote fire as an ecosystem process. | Develop & implement fire management plan. | Both parks recognize the ecological role of fire and have objectives to manage fire effectively within their ecosystems. | 5 |  |
|  | To manage the role of keystone species. | Key species reintroductions (Black rhino, Lichtenstein's Hartebeest). | KNP focuses on managing existing keystone species, while GNP emphasizes reintroducing key species, both contributing to biodiversity conservation. | 4 |  |
|  | To ensure suitable habitat exists for small predators. | No direct matching objective found. | KNP has a specific focus on small predator habitats, which is not explicitly addressed in GNP's listed objectives. | 0 |  |
|  | To strive for ecosystem health and resilience in park river systems. | Clarify ecological & threat monitoring (ETM) priorities; collect baseline & monitoring data. | KNP's goal for river system resilience aligns with GNP's broader ecological monitoring efforts, which can encompass river ecosystems. | 4 |  |
|  | To map the distribution of alien species inside the park and in priority neighboring areas, and implement effective monitoring to determine trends in the status of invasions and efficacy of control programmes. | Assess & mitigate invasive species threats. | Both objectives focus on addressing invasive species through assessment and mitigation strategies. | 5 |  |
|  | To develop a proactive relationship with safety and security authorities and alliance partners to assure quick and deliberate safety and security response actions. | Work with ZRP & judiciary to improve prosecutions outcomes. | Both parks aim to enhance safety and security through collaboration with law enforcement and judicial systems. | 5 |  |
|  | To provide adequate control over access to and movement within the park whilst ensuring and maintaining a safe and secure environment for both staff and visitors. | Provide adequate control over access to and movement within the park whilst ensuring and maintaining a safe and secure environment for both staff and visitors. | Both objectives align in promoting sustainable resource use through cooperative arrangements and legal frameworks. | 5 |  |
|  | To promote fire safety and reduce wildfire risk; develop & implement fire management plan. | Develop & implement fire management plan. | Both parks recognize the ecological role of fire and have objectives to manage fire effectively within their ecosystems. | 5 |  |
